# Thymic Carcinoma Invading Bilateral Internal Thoracic Artery Bypass Grafts: Diffuse Stenosis Suggesting Extravascular Compression

**DOI:** 10.1016/j.jaccas.2024.103220

**Published:** 2025-02-26

**Authors:** Minh Thien Nguyen, Masataka Kajiwara, Satoshi Yamamoto, Haruki Matsunaga, Toshio Katagiri, Takashi Fujimura, Tadashi Yamamoto, Manabu Hisahara, Yuji Hirai, Hiroyuki Tsutsui

**Affiliations:** a Department of Cardiovascular Medicine, Kouhoukai Takagi Hospital, Fukuoka, Japan; b Department of Thoracic Surgery, Kouhoukai Takagi Hospital, Fukuoka, Japan; c Department of Cardiovascular Surgery, Kouhoukai Takagi Hospital, Fukuoka, Japan

**Keywords:** coronary artery bypass graft surgery, internal thoracic artery graft, mediastinal mass, thymic carcinoma

## Abstract

Thymic carcinoma, a rare but aggressively growing malignancy that tends to invade nearby structures, requires early intervention and is often associated with poor prognosis. A 73-year-old man presented with vague chest pain. Imaging studies coincidently revealed an anterior mediastinal mass invading bilateral internal thoracic artery bypass grafts. Angiography showed diffuse stenosis due to tumor compression and numerous feeding arteries. Positron emission tomography scans led to the clinical diagnosis of thymic carcinoma, stage IVb. We reported an extremely rare and complicated case of unresectable thymic carcinoma invading coronary artery bypass grafts, aiming to discuss the complex features challenging the diagnostic approach and management of the disease. Diffuse stenosis on angiography may suggest extravascular compression by coincident mediastinal tumors. Complicated cases of mediastinal neoplasm with invasion of vital structures require individualized diagnostic approaches and treatment strategies.

## Graphical Abstract

**Figure:**
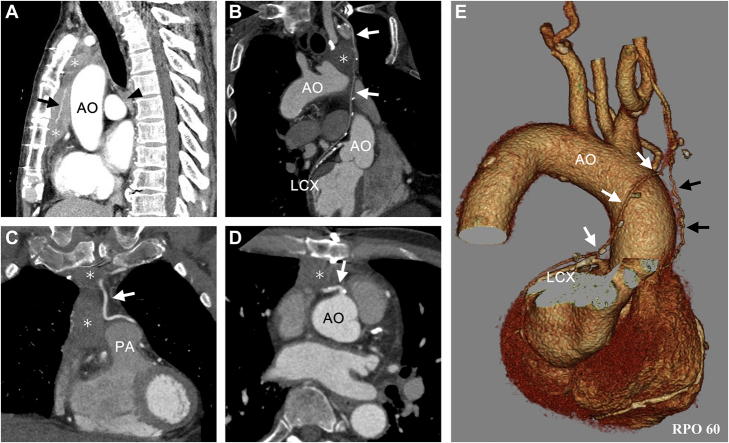

## History of Presentation

A 73-year-old man presented with 1 month of persistent dry cough, mild fatigue, and vague and constant chest pain right behind the sternum. Physical examination, chest X-ray, and electrocardiogram were unremarkable.

**Take-Home Messages**

• Complicated cases of mediastinal neoplasm with invasion of vital structures, such as coronary bypass grafts, require individualized diagnostic approaches and treatment strategies.
• Diffuse stenosis of the coronary bypass graft may suggest extravascular compression by coincident mediastinal tumors such as thymic carcinoma.

## Past Medical History

The patient underwent coronary artery bypass graft (CABG) surgery with bilateral internal thoracic artery (ITA) grafts 15 years prior due to exertional angina pectoralis. His coronary angiography at that time revealed severe stenosis, left main coronary trunk of 90%, proximal left anterior descending artery (LAD) of 99%, and proximal left circumflex artery (LCX) of 50% with a left main coronary trunk true bifurcation lesion. CABG was performed with the left internal thoracic artery graft to LAD (LITA-LAD) and the right internal thoracic artery graft to LCX (RITA-LCX). The postoperative course was uneventful, and follow-up coronary computed tomography (CT) angiography at 2 and 6 years revealed patent grafts and no other abnormalities. Comorbidities included hypertension and uncontrolled diabetes mellitus.

## Investigations

**Figure 1:**
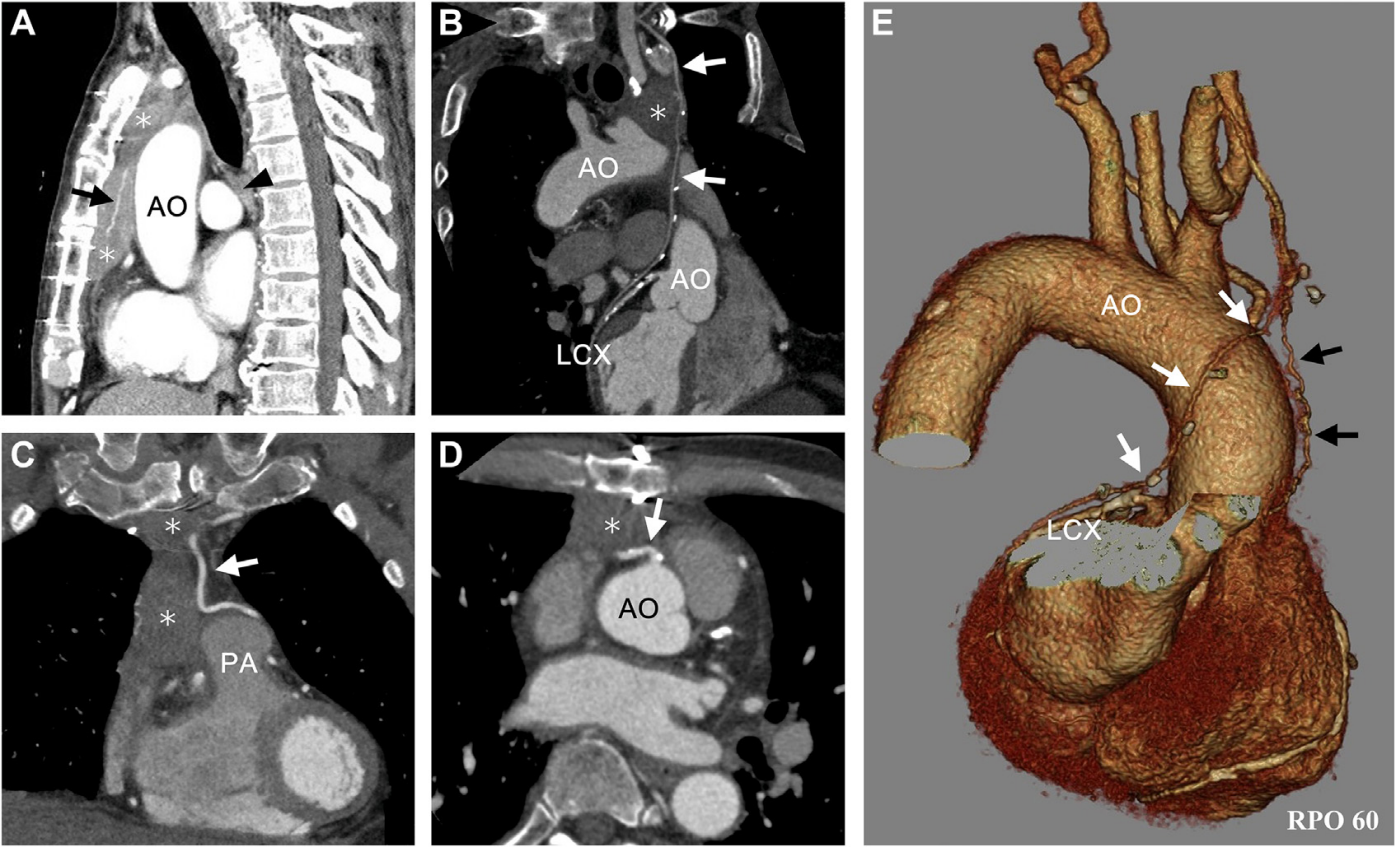
Contrast-Enhanced CT Images (A) A complex infiltrating anterior mediastinal mass (asterisks) behind the sternum. The smaller upper part and the larger lower part, wrapping around a large feeding vessel (arrow). An enlarged subcarinal lymph node suggesting lymph node metastasis (arrowhead) was observed. (B) Proximal right internal thoracic artery graft (white arrows) infiltrated by the tumor upper part (asterisk). (C) The tumor (asterisks) medially invaded the left internal thoracic artery graft (white arrow). (D) The tumor (asterisk) infiltrated the proximal right coronary artery (white arrow) and pericardium. (E) Three-dimensional CT reconstruction image showed a large feeding artery (black arrows) from the RITA (white arrows). AO = aorta; CT = computed tomography; LCX = left circumflex artery; PA = pulmonary artery.

Biochemical and hematologic tests revealed no abnormalities except hemoglobin A1c of 8.9%. Transthoracic echocardiography showed a mild reduced ejection fraction of 45% and diffuse mild hypokinesis. Subsequent CT imaging of lung fields was unremarkable but incidentally revealed a complex anterior mediastinal mass with irregular contours behind the sternum. The mass was grossly divided into 2 continuous parts: the smaller upper part (2.8 × 2.1 × 2.7 cm) at the aortic arch level and the larger lower part (3.2 × 2.3 × 6.2 cm) at the aortic root level. The tumor adhered to the sternum, proximal right coronary artery, pericardium, aorta root, and pulmonary trunk. Contrast-enhanced CT defined a non-encapsulated mass with a hypo-vascularized inhomogeneous appearance, medially invading bilateral ITA grafts along their course in the anterior mediastinum (Figure 1).

**Figure 2:**
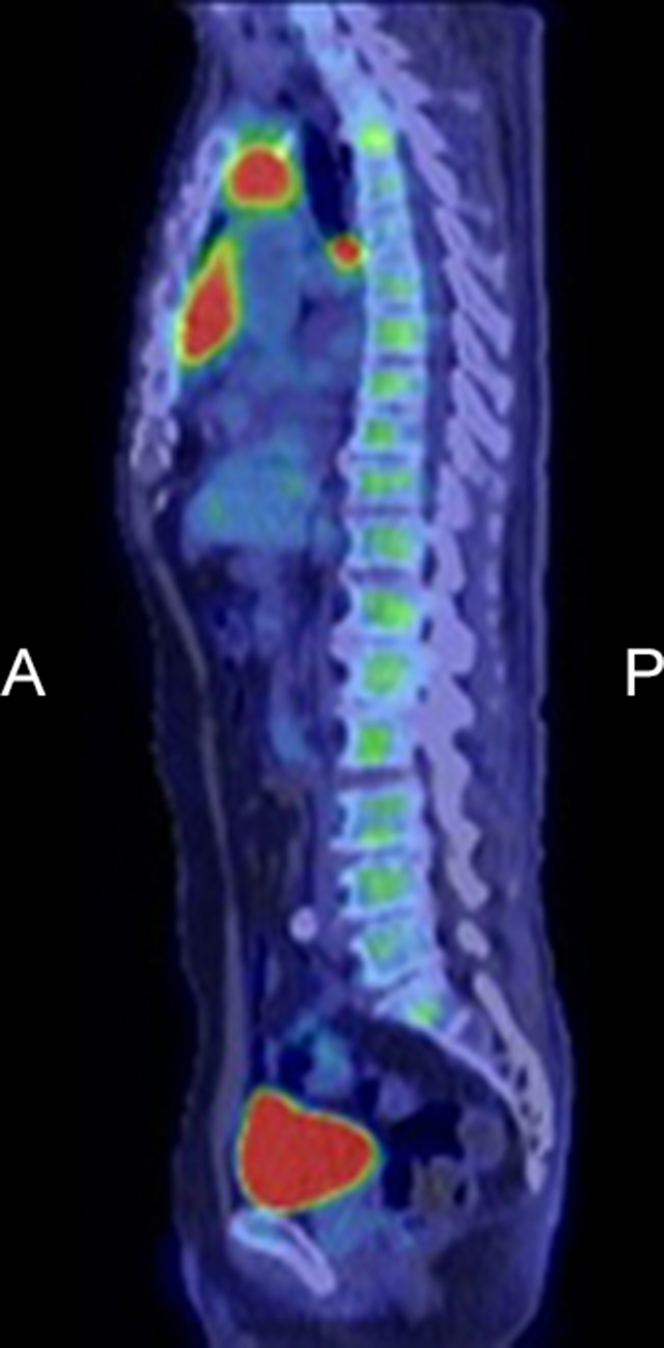
PET/CT Image Significantly increased uptakes in the upper part (SUV max = 5.78) and lower part (SUV max: 6.63) of the tumor, and enlarged subcarinal lymph node (SUV max = 7.37). T/M ratio = 5.602. A = anterior; P = posterior; PET/CT = positron emission tomography integrated with computed tomography; SUV = standardized uptake value; T/M ratio = ratio of peak SUV max of the tumor to the mean SUV of the mediastinum at the level of the aortic arch.

Tumor marker studies indicated high levels of serum cytokeratin fragment (2.0 ng/mL) and squamous cell carcinoma antigen (20 ng/mL) but negative in carcinoembryonic antigen, human chorionic gonadotropin, alpha-fetoprotein, and soluble interleukin-2 receptor. A positron emission tomography (PET) with 2-deoxy-2-[fluorine-18] fluoro-D-glucose (18F-FDG) integrated with CT scan revealed significantly increased FDG uptakes in the upper part, the lower part, and enlarged subcarinal lymph node, suggesting lymph node metastasis (Figure 2). A whole-body PET/CT scan detected no abnormally increased uptake in other organs. Based on these findings, the clinical diagnosis of thymic carcinoma, TMN classification of T4N2M0, and staging IVb was made.^1^

**Figure 3:**
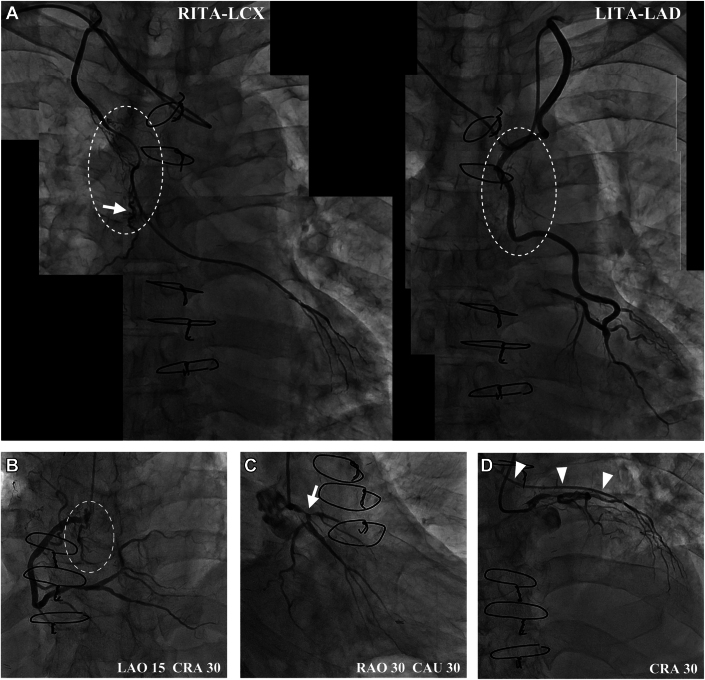
Coronary Artery and Bypass Graft Angiographic Images (A) Patent grafts with diffuse stenosis of the middle course of RITA through the upper mass. A large solitary feeding artery from RITA (arrow) and numerous other small tortuous vessels (intermittent outlines) nourishing the tumor (Videos 1 and 2). (B) CAG showed 50% stenosis of proximal right coronary artery, poor mobility with heartbeats and neovascularization toward the tumor (intermittent outlines) (Video 3). (C) CAG showed a true bifurcation lesion of the native left main coronary trunk (arrow) (Video 4). (D) RITA graft had reversal flow (arrowheads) from the native LCX (Video 5). CAG = coronary angiography; CAU = caudal; CRA = cranial; LAD = left anterior descending artery; LAO = left anterior oblique; LCX = left circumflex artery; LITA = left internal thoracic artery; RAO = right anterior oblique; RITA = right internal thoracic artery.

Coronary angiography (Figure 3) showed patent LITA and RITA, diffuse stenosis of the middle course of RITA, and numerous feeding arteries derived from bilateral grafts toward the tumor (Videos 1 and 2). The proximal right coronary artery angiography showed poor mobility with heartbeats and neo-vascularization toward the thymic carcinoma (Video 3). Because the RITA graft had reversal flow from the native LCX, RITA stenosis was considered to be significant (Videos 4 and 5).

Transthoracic CT-guided needle biopsy, mediastinoscopy, and surgical resection of the tumor via midline sternotomy were deferred due to the high risk of damage to bilateral ITA grafts, severe mediastinal adhesion post-CABG, and uncontrolled diabetes.

## Outcome and Follow-Up

Treatment choices, including radiation therapy and chemotherapy, as well as their risks and benefits, were discussed with the patient. After multidisciplinary discussion and careful consideration, the patient refused proposed invasive therapies and accepted palliative care only.

Although his symptoms persist, he was otherwise performing well during the subsequent 6-month follow-up.

## Discussion

An anterior mediastinal mass in a post-CABG patient gives a diagnosis of thymoma, lymphoma, germ-cell tumor, and graft pseudoaneurysm. The latter is uncommon and mostly seen in CABG with saphenous vein graft. Unlike thymoma, a benign encapsulated tumor, thymic carcinoma exhibits more aggressive features and is often adherent to nearby structures such as pericardium, lung, and great vessels. Squamous cell carcinoma is the most common histological type of thymic carcinoma, often suggested by positive cytokeratin fragment and squamous cell carcinoma antigen tumor markers, also seen in this case.^2^ Histologic assessment via CT-guided transthoracic core needle biopsy or mediastinoscopy was deferred due to the presence of the ITA grafts passing through the tumor, which might impose a high risk of bleeding and impaired coronary blood flow. Instead, the PET/CT scan was performed because it is minimally invasive, and studies have proved its superiority in diagnosing thyroid carcinoma.^3^^,^^4^ A whole-body PET scan helped detect metastasis in a deep intrathoracic lymph node and ruled out malignancies from other organs that may metastasize to the mediastinum.

Without treatment, thymic carcinomas are associated with a poor prognosis; however, the optimal choice of treatment for this patient is challenging. Owing to the uncommon nature of thymic carcinoma, evidence on treatment therapies is limited and controversial, especially when complicated by tumor invasion of critical structures. In this case, surgical resection via redo median sternotomy was complicated and risky due to the tumor encroachment to the mediastinal structure, the course of ITA graft within the tumor, the state of metastasis, and uncontrolled diabetes mellitus. Reoperative cardiac surgery itself is already associated with a significant risk of complications and poor outcomes.^5^ Complete resection was unlikely; the risk of a debulking surgery outweighed its benefit. In addition, tumor resection means sacrificing the ITA grafts, which requires preoperative security of coronary blood flow and possible inoperative revascularization.

The first-line treatment for unresectable thymic tumors is radiotherapy, which may cause graft stenosis or damage caused by irradiation. However, graft flow will inevitably be impaired without treatment due to gradual tumor invasion. Prophylactic percutaneous coronary intervention was challenging and risky owing to severe stenosis of the left coronary artery bifurcation. Moreover, metal stents in the irradiation field may induce overexposure to the surrounding tissues, especially coronary arteries. Stent placement to bilateral ITA grafts was a possible percutaneous coronary intervention option; however, stent degeneration owing to tumor infiltration may occur, and limited evidence is established regarding this treatment. Platinum-based chemotherapy regimens are also feasible, but the evidence is controversial.

## Conclusions

Previous case reports have documented benign thymomas involving coronary bypass grafts.^6^, ^7^, ^8^ In the present case, we report an extremely rare and complicated case of unresectable thymic carcinoma invading ITA grafts to discuss the diagnostic approaches and management strategies of future complicated cases like this.

## Funding Support and Author Disclosures

The authors have reported that they have no relationships relevant to the contents of this paper to disclose.

## Appendix

Video 1 Angiography of Right Internal Thoracic Artery

Video 2 Angiography of Left Internal Thoracic Artery

Video 3 Angiography of Right Coronary Artery

Video 4 Angiography of Left Coronary Artery (RAO-CAU)

Video 5 Angiography of Left Coronary Artery (CRA)

## References

[1] Goldstraw P., Chansky K., Crowley J. (2016). The IASLC Lung Cancer Staging Project: Proposals for revision of the TNM stage groupings in the forthcoming (eighth) edition of the TNM classification for lung cancer. J Thorac Oncol.

[2] Suster S. (2005). Thymic carcinoma: update of current diagnostic criteria and histologic types. Semin Diagn Pathol.

[3] Marom E.M. (2013). Advances in thymoma imaging. J Thorac Imaging.

[4] Seki N., Sakamoto S., Karube Y., Oyaizu T., Ishihama H., Chida M. (2014). ^18^F-fluorodeoxyglucose positron emission tomography for evaluation of thymic epithelial tumors: utility for World Health Organization classification and predicting recurrence-free survival. Ann Nucl Med.

[5] Yaku H., Doi K. (2014). Redo coronary artery bypass grafting. Gen Thorac Cardiovasc Surg.

[6] Dubique J.Y., Turbendian H., Chu D. (2017). Neovascularization of thymoma from left internal mammary artery bypass graft. Ann Thorac Surg.

[7] Jaklitsch M.T., Byrne J.G., Mery C. (1999). Thymoma encasing last patent vein graft to the heart. J Thorac Cardiovasc Surg.

[8] Thomas P.A., Collart F., Doddoli C., Gariboldi V., Moulin G. (2006). Nourishing vascularization of a thymoma issued from a left internal thoracic artery graft. J Thorac Cardiovasc Surg.

